# Therapeutic efficacy of artesunate–amodiaquine and artemether–lumefantrine for the treatment of uncomplicated falciparum malaria in Chad: clinical and genetic surveillance

**DOI:** 10.1186/s12936-023-04644-w

**Published:** 2023-08-23

**Authors:** Mahamat Souleymane Issa, Marian Warsame, Moussa Hassane Taisso Mahamat, Issakha Diar Mahamat Saleh, Kodbsse Boulotigam, Honoré Djimrassengar, Ali Haggar Issa, Ousmane Abdelkader, Manah Hassoumi, Mbanga Djimadoum, Cécile Doderer-Lang, Jean Bosco Ndihiokubwayo, Charlotte Rasmussen, Didier Menard

**Affiliations:** 1 Chad National Malaria Control Programme, N’Djamena, Chad; 2 School of Public Health and Community Medicine, University of Gothenburg, Gothenburg, Sweden; 3 Faculty of Science and Human Health, University of N’Djamena, N’Djamena, Chad; 4 World Health Organization, N’Djamena, Chad; 5 Ecole Nationale des Agents Sanitaires et Sociaux (ENASS), N’Djamena, Chad; 6 Ministry of Health, Public and Human Solidarity, N’Djamena, Chad; 7 Hôpital de l’Amitié Tchad-Chine, N’Djamena, Chad; 8 Institute of Parasitology and Tropical Diseases, UR7292 Dynamics of Host-Pathogen Interactions, Université de Strasbourg, 67000 Strasbourg, France; 9 World Health Organization, Geneva, Switzerland; 10 Malaria Genetics and Resistance Unit, INSERM U1201, Institut Pasteur, Université Paris Cité, 75015 Paris, France; 11 Malaria Parasite Biology and Vaccines Unit, Institut Pasteur, Université Paris Cité, 75015 Paris, France; 12 Laboratory of Parasitology and Medical Mycology, CHU Strasbourg, 67000 Strasbourg, France

**Keywords:** Malaria, *Plasmodium falciparum*, Artesunate–amodiaquine, Artemether–lumefantrine, Artemisinin resistance, *pfKelch13*, *Pfcrt*, *Pfmdr-1*, *Pfdhfr*, *Pfdhps*, *Pfhrp-2* deletion, HRP2-based RDT, Chad

## Abstract

**Background:**

Artesunate–amodiaquine (AS–AQ) and artemether–lumefantrine (AL) are the currently recommended first-and second-line therapies for uncomplicated *Plasmodium falciparum* infections in Chad. This study assessed the efficacy of these artemisinin-based combinations, proportion of day 3 positive patients, proportions of molecular markers associated with *P. falciparum* resistance to anti-malarial drugs and variable performance of HRP2-based malaria rapid diagnostic tests (RDTs).

**Methods:**

A single-arm prospective study assessing the efficacy of AS–AQ and AL at three sites (Doba, Kelo and Koyom) was conducted between November 2020 to January 2021. Febrile children aged 6 to 59 months with confirmed uncomplicated *P. falciparum* infection were enrolled sequentially first to AS–AQ and then AL at each site and followed up for 28 days. The primary endpoint was PCR-adjusted adequate clinical and parasitological response (ACPR). Samples collected on day 0 were analysed for mutations in *pfkelch13*, *pfcrt*, *pfmdr-1*, *pfdhfr, pfdhps* genes and deletions in *pfhrp2/pfhrp3* genes.

**Results:**

By the end of 28-day follow-up, per-protocol PCR corrected ACPR of 97.8% (CI 95% 88.2–100) in Kelo and 100% in Doba and Kayoma were observed among AL treated patients. For ASAQ, 100% ACPR was found in all sites. All, but one patient, did not have parasites detected on day 3. Out of the 215 day 0 samples, 96.7% showed *pfkelch13* wild type allele. Seven isolates carried nonsynonymous mutations not known to be associated artemisinin partial resistance (ART-R). Most of samples had a *pfcrt* wild type allele (79% to 89%). The most prevalent *pfmdr-1* allele detected was the single mutant 184F (51.2%). For *pfdhfr* and *pfdhps* mutations, the quintuple mutant allele N51I/C59R/S108N + G437A/540E responsible for SP treatment failures in adults and children was not detected. Single deletion in the *pfhrp2* and *pfhrp3* gene were detected in 10/215 (4.7%) and 2/215 (0.9%), respectively. Dual *pfhrp2/pfhrp3* deletions, potentially threatening the efficacy of HRP2-based RDTs, were observed in 5/215 (2.3%) isolates.

**Conclusion:**

The results of this study confirm that AS–AQ and AL treatments are highly efficacious in study areas in Chad. The absence of known *pfkelch13* mutations in the study sites and the high parasite clearance rate at day 3 suggest the absence of ART-R. The absence of *pfdhfr/pfdhps* quintuple or sextuple (quintuple + 581G) mutant supports the continued use of SP for IPTp during pregnancy. The presence of parasites with dual *pfhrp2/pfhrp3* deletions, potentially threatening the efficacy of HRP2-based RDTs, warrants the continued surveillance.

*Trial registration* ACTRN12622001476729

**Supplementary Information:**

The online version contains supplementary material available at 10.1186/s12936-023-04644-w.

## Background

Provision of effective treatment, using artemisinin-based combination therapy (ACT), fast-acting artemisinin derivatives partnered with longer-acting partner drugs, is a critical component of recommended malaria interventions [1]. The World Health Organization (WHO) recommends artemether–lumefantrine (AL), artesunate–amodiaquine (AS–AQ), artesunate–mefloquine (AS–MQ), dihydroartemisinin–piperaquine (DHA–PPQ), artesunate–sulfadoxine/pyrimethamine (AS–SP) and artesunate–pyronaridine (AS–PY) for the treatment of uncomplicated falciparum malaria infection [2]. The WHO also recommends intermittent preventive treatment with SP during pregnancy (IPTp-SP) and Seasonal Malaria Chemoprevention (SMC) using SP + AQ to protect children during the season of greatest risk in falciparum endemic areas in Africa [1].

Resistance of *Plasmodium falciparum* to anti-malarial drugs remains a threat to effective case management and undermines the global efforts to control and eliminate the burden of malaria. Artemisinin partial resistance (ART-R), defined as delayed clearance of parasitaemia (parasite half-life > 5 h or day 3 parasitaemia) following ACT or artemisinin monotherapy, emerged first in Southeast Asia [3, 4]. Non-synonymous mutations in the propeller region of the *P. falciparum kelch13* (*pfkelch13*) gene have been documented to be associated with ART-R [5]. Recent emergence and expansion of validated *pfkelch13* mutations in Rwanda (R561H), Uganda (A675V or C469Y) associated with delayed parasite clearance are of great concern [6, 7] and calls for frequent monitoring of ACT efficacy, including clearance of parasitaemia, and *pfkelch13* mutations in Africa, as recommended by the WHO [8].

The SP combination inhibits the enzymes dihydrofolate reductase (DHFR) and dihydropteroate synthase (DHPS), which are involved in the folate pathway of nucleic acid synthesis of the parasite. Unfortunately, mutations in the parasite genes *pfdhfr* (codons 51, 59, 108 and 164) and *pfdhps* (codons 437, 540, 581 and 613) were found to confer resistance to pyrimethamine and sulfadoxine, respectively [9–11]. The *pfdhfr* triple mutant (N51I, C59R, S108N) combined with the *pfdhps* double mutant (A437G, K540E) have been associated with in vivo resistance to SP [12, 13] and the sextuple mutant (quintuple mutant with an additional mutation in codon 581 of the *pfdhps* gene) with clinical failure and loss of SP protection when the prevalence of this mutant exceeds 10% [14–17]. However, several studies suggest that although the use of IPTp-SP often results in the selection and increased prevalence of resistance-associated genetic mutations, this does not necessarily lead to a decrease in malaria prevention efficacy [18].

In Chad, malaria accounted for 43% of out-patient attendances, 44% of hospitalizations and 60% of hospital deaths among children in 2018 [19]. Since 2005, AS–AQ and AL are recommended first and second line treatments, respectively, for the treatment of uncomplicated *P. falciparum* malaria. To control the burden of malaria in the country, IPTp-SP is a part of the recommended interventions to control malaria among the pregnant women [20]. A study conducted in 2015 reported high cure rate (100%) with AS–AQ and absence of mutation in *pfkelch13* gene among under-five children with uncomplicated falciparum malaria [21].

The aim of the current study was to monitor the therapeutic efficacy of AS–AQ and AL for the treatment of uncomplicated *P. falciparum* malaria in children and assess the proportion of day3 positive patients. Mutations in *pfkelch13*, *pfcrt*, *pfmdr-1*, *pfdhfr*, *pfdhps* associated with *P. falciparum* resistance to anti-malarial drugs along with deletions in *pfhrp2/pfhrp3* involved in variable performance of HRP2-based malaria rapid diagnostic tests (RDTs) were also investigated in isolates collected prior anti-malarial treatment and those from patients experiencing recurrences.

## Methods

### Study design and areas

**Fig. 1:**
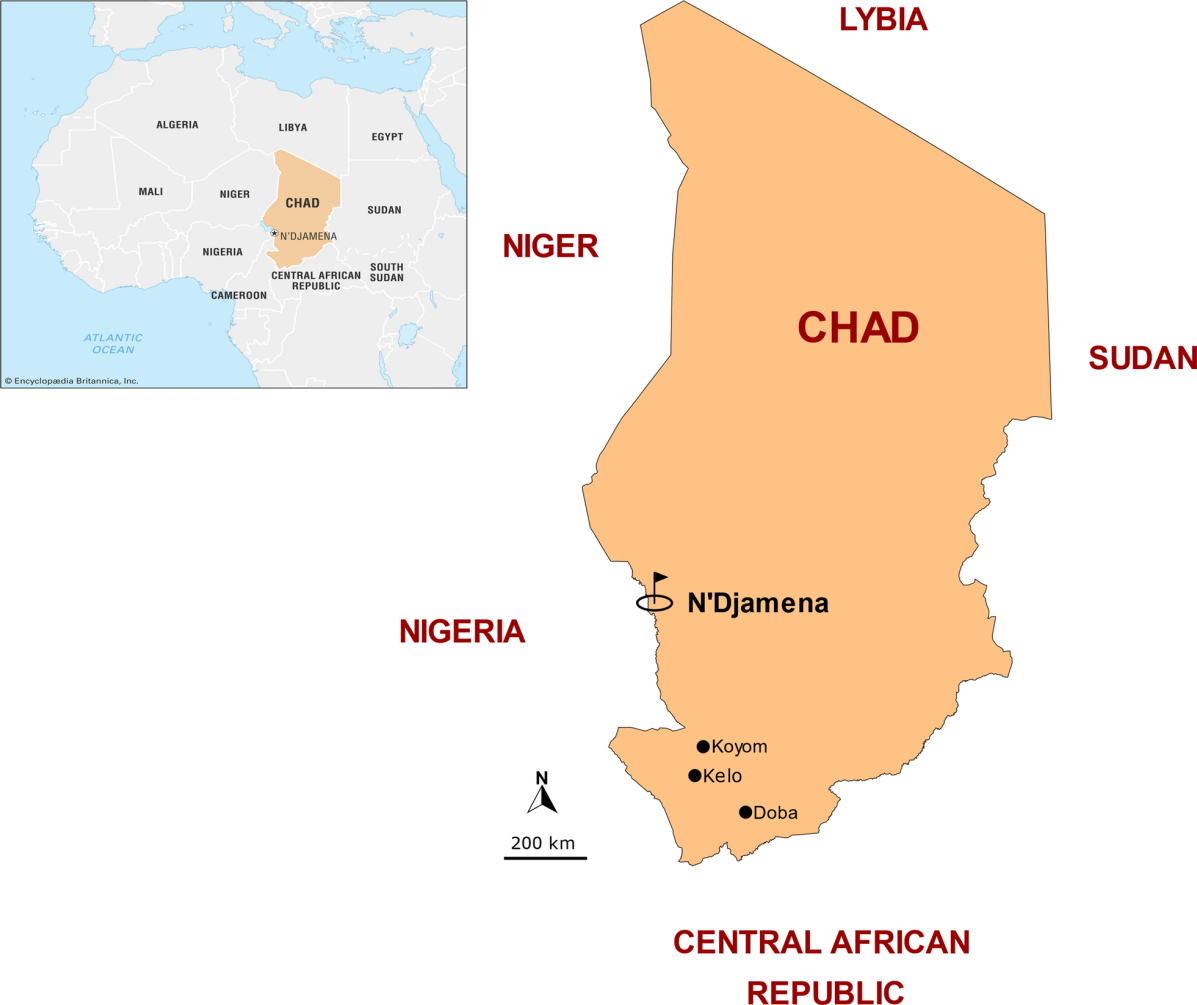
Location of the study sites, Chad, 2022–2023

The study used one arm prospective design to assess the efficacy of AL and AS–AQ for the treatment of uncomplicated falciparum malaria among children aged 6 to 59 months from November 2020 to January 2021. The study sites were the (i) Gaki health center in Doba town (8° 40′ N, 16° 51′ E) in Pende department of Logone Oriental, (ii) Hindina health center in Kelo town in West Tandjile department of Tandjile province and (iii) Boubou health center in Koyom town in Mayo Boneye department of Mayo Kebbi Est province. Malaria transmission in the study areas occurs from June to October and *P. falciparum* is the predominant species [19] (Fig. 1).

### Sample size estimation

Sample size estimates assumed 5% of treatment failure with ASAQ or AL treatment in the study sites. At a confidence level of 95% and an estimate precision of 10%, a minimum sample size of 50 patients was required. With a 20% increase to allow for loss to follow-up and withdrawals during the 28-days of follow-up, 60 patients were targeted per drug and per site.

### Recruitment and treatment procedures

Children attending the study health clinics between November 2020 and January 2021 were screened for eligibility criteria, and were enrolled, after obtaining consent from parents/guardians, if they met the study inclusion criteria: age 6 to 59 months, axillary temperature of ≥ 37.5 °C or history of fever during the past 24 h; *P. falciparum* mono-infection with parasitaemia of 2000 to 200,000 asexual parasites/µl by microscopy. Children presenting with signs or symptoms of severe falciparum malaria, non-falciparum species or severe malnutrition, third party administration of anti-malarial drugs and febrile conditions due to diseases other than malaria, were not recruited and received appropriate treatment and care according to national guidelines. Children who developed danger signs (convulsions, lethargy, inability to drink or breast feed, repeated vomiting, inability to stand/sit due to weakness) or severe malaria were referred to receive parenteral artesunate with dose regiments according to the national treatment guidelines.

Children were enrolled sequentially first to AS–AQ (Sanofi, lot number 8MA153) and then AL (Cipla, lot number ID01029) at each site. Children treated with AS–AQ received artesunate 4 mg/kg + amodiaquine 10 mg/kg once daily over 3 consecutive days. ASAQ tablets were given based on the following weight bands: one tablet of 25 artesunate + 67.5 amodiaquine for 4.5 to < 9 kg body weight, one table of 50 artesunate + 135 amodiaquine for 9–< 18 kg body weight, one tablet of 100 artesunate + 270 amodiaquine for 18 to < 36 kg body weight. The AL treated group received twice daily doses of the ACT for 3 days based on weight bands: one tablet for 5–14 kg and two tablets for 15–24 kg. Artemether–lumefantrine was administered with milk when possible. All treatment doses were given under direct observation by the study team and patients were observed for 30 min. If the first dose was vomited, treatment was re-administered. If vomited second time, the child was given parenteral artesunate according to the national treatment guidelines and the patient was withdrawn from the study. WHO/HQ provided the study drugs. Enrolled children were monitored for 28 days, and clinical and parasitological assessment were done at scheduled visits (days 1, 2, 3, 7, 14, 21 and 28) or unscheduled visits if children felt ill. Patients who did not return for follow-up were visited at home.

### Malaria microscopy

Thick and thin blood smears were obtained through finger prick to detect the presence of *P. falciparum* and estimate parasite density at day 0 (before inclusion) and at each scheduled (days 1, 3, 7, 14, 21 and 28) or unscheduled visits. The blood smears were dried, Giemsa-stained and examined under light microscopy at 100× magnification. Parasite count and density (per μl blood) were determined using WHO procedure [22]. If the two readings were discordant, in terms of difference in parasite positivity, parasite species or parasite density above 50%, the slide was read by a third microscopist.

### Treatment responses

The treatment outcomes by day 28 were classified, using on the WHO 2009 protocol [22], as adequate clinical and parasitological response (ACPR), early treatment failure (ETF), late parasitological failure (LPF), and late clinical failure (LCF). PCR analysis was performed to distinguish between a true recrudescence due to treatment failure and episodes of reinfection.

### Parasite genotyping to differentiate recrudescence from re-infection

Filter paper blood samples collected from each patient on day 0 and on the day of parasite recurrence (from day 7 onward) were stored in individual plastic bags with desiccant and protected from light, moisture and extreme temperature until analysis. Each dried blood spot was cut out sterilely and placed in an Eppendorf tube. Parasite DNA was extracted by using the 96-well format protocol developed by Zainabadi et al. [23]. The eluted DNA was then quantified by fluorometric quantitation (Qubit, Thermo Fischer), adjusted to 20 ng/μl and stored at − 20 °C for later use. Paired DNA from patients with recurrent parasites (day-0 and day of recurrence) were genotyped using nested polymerase chain reaction (PCR) targeting the highly polymorphic genes *msp1, msp2* and the microsatellite marker poly-α [24], according to the recent WHO recommendation [25]. All markers were assessed systematically. The fragment sizes were estimated by capillary electrophoresis (Fragment analyzer, Agilent) and the cut-off settings for PCR artefacts and stutter peaks was defined for peaks < 10% of the low and upper control bands. The bins used to define a match were ± 10 bp for *msp1/msp2*, and ± 5 bp for *poly-α.* Genotyping data were compared with the former genotyping approach assessing *msp1*, *msp2* and *glurp* [26]. The bins used to define a match was ± 20 bp for *glurp*. The WHO/MMV decision algorithm was used to define PCR-adjusted clinical efficacy rates. Recrudescence was defined as a genotype that had already been detected in the blood sample taken before treatment (i.e., at least one allele is shared at day 0 and day of parasite recurrence at all three loci). A new infection was defined as the absence of a shared allele between day 0 and day of parasite recurrence at any of the three loci.

### Molecular markers

Day 0 DNA were analysed for the presence of point mutations in the *pfkelch13* gene (codons 430–720) associated with ART-R [5], the *pfcrt* (at codons 72–76, 93, 97, 145, 218, 343, 350 and 353) and *pfmdr-1* (at codons 86, 184, 1034, 1042 and 1246) genes associated or suspected to be associated with 4-aminoquinolines and aminoalcohol resistance [27], the *pfdhfr* (at codons 51, 59, 108, 164) and *pfdhps* (at codons 431, 436, 437, 540, 581, 613) genes linked to pyrimethamine and sulfadoxine resistance [27]. *hrp2* and *hrp3* deletions that can cause false-negative results with HRP2-based rapid diagnostic tests (RDTs) were also screened [28].

Amplicons from targeted sequences were generated using multiplexing nested PCR assays using indexed primers that themselves contain specific tags (barcodes) consisting of individual 8-base indices, specific to the sample and adapter sequences (14 or 15 bases) that allow the final PCR product to bind to the sequencing flow cell (Table 1). A total of 4 μl of PCR reactions from each sample were mixed in one pool (96 samples) to increase the sample volume and minimize sample quantity for downstream protocol steps. For each pool, the amplicons were then purified with the AMPure XP beads (Beckman Coulter), according to manufacturer’s protocol to eliminate dNTPs, salts, primers and primer dimers. The quality of purified PCR products was assessed by analysing eluates containing the purified amplicons on a Fragment analyzer (Agilent). DNA concentration of pooled fragments was assessed by fluorometric quantitation (Qubit, Thermo Fischer). The pooled libraries were denatured with NaOH to a final concentration of 0.1 N, diluted with hybridization buffer before running the sequencing. Sequencing was performed using the MiSeq v2 reagents using the 300-cycle kit (Illumina) according to the manufacturer’s recommendations. The raw sequences were demultiplexed and quality trimmed at a phred score of 30. The primer sequences were trimmed from the 5′-end of the sequences, to avoid primer bias in the sequenced fragments. Base calling was performed by comparing reads with a custom database, consisting of the 3D7 reference sequence. Bioinformatic analysis were performed using the CLC Genomics Workbench 22 software (Qiagen). Laboratory reference parasite strains (Dd2, 7G8, HB3 and a Cambodian culture-adapted strain), with known alleles in each gene were used as controls.

**Table 1:**
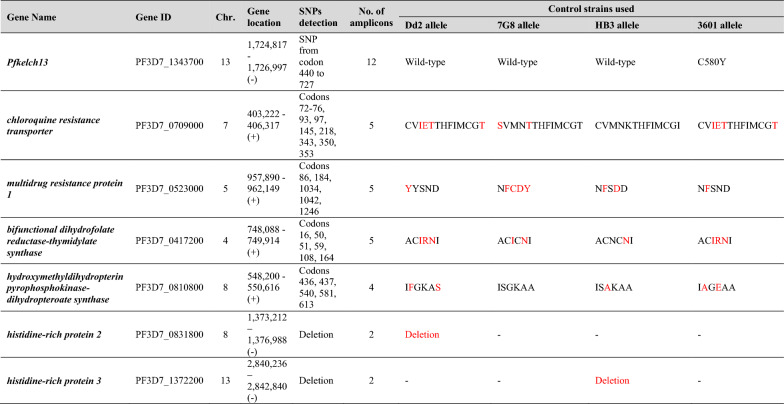
List of the targeted regions, number of amplicons generated by multiplexing nested PCR assays and list of the alleles of the parasite strains used as controls

Position of amino acid corresponds to codons 72, 73, 74, 75, 76, 93, 97, 145, 218, 343, 350, 353 and 356 for pfcrt, to codons 86, 184, 1034, 1042 and 1246 for pfmdr-1, to codons 16, 50, 51, 59, 108, 164 for dhfr and to codons 431, 436, 437, 540, 581, 613 for dhps

### Ethical considerations

Before study implementation, it obtained approval from the National Ethics Committee of Chad and the WHO ERC. The study objectives, its benefits and potential risks and its procedures were explained to the parents/guardians of the potential study children. Children were only included in the study if their parents or guardians gave written consent. If the parent or guardian cannot read or write, a witness chosen by the parent/guardian of the child consigned the consent form.

### Statistical analysis

WHO excel software (WHO excel software programme (http://www.who.int/malaria/publications/atoz/9789241597531/en/) was used to double enter and analyse the data. Both per-protocol and Kaplan Meier analysis were used to evaluate the treatment outcomes. Patients who were lost or withdrawn or had new infection during the follow-up were excluded from the per-protocol analysis while these cases were censored last day the patient was follow-up, withdrawn or experienced new infection. Patients with indeterminate PCR results were excluded from both per-protocol and KM analysis. Percentages, mean, standard deviation and range were presented. Categorical variables were compared using Chi-square or Fisher exact test, and t-test for continuous variables. A p-value of < 0.05 was considered statistically significant.

## Results

### Clinical outcomes

Table 2 summarizes the baseline characteristics of the study children by site and drug. In total, 114 and 101 children were recruited for AL and AS–AQ, respectively. Due to the late start of the study in the transmission season, the targeted sample per drug (n = 60) in each site was not reached. There was no significant difference in the baseline patient profile between the sites and between treatment groups.

**Table 2:** Baseline characteristics of the study patients

	Artemether–lumefantrine			Artesunate–amodiaquine		
	Doba	Kelo	Kayoma	Doba	Kelo	Kayoma
Number of patients	31	48	35	33	33	35
Males, %	61%	56%	40%	58%	58%	60%
Age (years)						
Mean (sd*)	3.3 (1.6)	2.6 (1.4)	3.2 (1.5)	2.9 (1.5)	2.7 (1.7)	2.7 (1.5)
Range (min–max)	0.5–5	0.5–5	0.5–5	0.5–5	0.5–5	0.5–5
Temperature (°C), day 0						
Mean (sd*),	38.1 (1.2)	38.1 (1.3)	38.6 (0.8)	38 (1.3)	38 (1.2)	38.3 (0.8)
Parasitaemia (µl), day 0						
Geometric mean	11,491	10,932	5073	13,909	12,524	4777
Range (min–max)	2000–20,000	2003–80,000	2000–40,000	2560–97,560	2000–90,000	2000–16,000

*Standard deviation

At the end of the 28 days follow up period, 111 (28 in Doba, 48 in Kelo and 35 in Kayoma) and 94 (30 in Doba, 41 in Kelo and 33 in Kayoma) patients in the AL and AS–AQ groups, respectively, reached the desired endpoints. Three patients in the AL and 7 in the AS–AQ groups were lost to follow-up or withdrawn, and consequently were excluded from the per-protocol analysis.

Per-protocol PCR uncorrected ACPR rate for AL were 100% (CI 95% 87.7–100) in Doba, 91.7% (CI 95% 80.0–97.7) in Kelo and 97.1% (CI 95% 85.1–99.9) in Kayoma, with an overall rate of 95.5% (CI 95% 89.8–98.5) (Table 3). For the AS–AQ treated groups, PCR uncorrected ACPR of 96.7% (CI 95% 82.8–99.9) in Doba, 100% in Kelo (CI 95% 88.8–100) and Kayoma (CI 95% 89.4–100) were observed with overall cure rate of 98.9% (CI 95% 94.2–100).

**Table 3:** PCR uncorrected treatment outcome

Treatment outcome	Artemether–lumefantrine						Artesunate–amodiaquine					
	Doba (n = 31)		Kelo (n = 48)		Kayom (n = 35)		Doba (n = 33)		Kelo (n = 33)		Kayom (n = 35)	
	n (%)	CI 95%	n (%)	95% CI	n (%)	95% CI	n (%)	CI 95%	n (%)	95% CI	n (%)	95% CI
*Per-protocol*												
ETF	0 (0)	0.0–12.3	0 (0)	0.0–7.4	0 (0)	0.0–10.0	0 (0)	0.0–11.6	0 (0)	0.0–11.2	0 (0)	0.0–10.6
LCF	0 (0)	0.0–12.3	0 (0)	0.0–7.4	0 (0)	0.0–10.0	0 (0)	0.0–11.6	0 (0)	0.0–11.2	0 (0)	0.0–10.6
LPF	0 (0)	0.0–12.3	4 (8.3)	2.3–20.0	1 (2.9)	0.1–14.9	1 (3.3)	0.1–17.2	0 (0)	0.0–11.2	0 (0)	0.0–10.6
ACPR	28 (100)	87.7–100	44 (91.7)	80.0–97.7	34 (97.1)	85.1–99.9	29 (96.7)	82.8–99.9	31 (100)	88.8–100	33 (100)	89.4–100
Total per-protocol	28		48		35		30		31		33	
Withdrawn/lost	3 (9.7)		0 (0)		0 (0)		3 (9.1)		2 (6.1)		2 (5.7)	
*Kaplan–Meier*												
Cure rate	100	NA	91.7	79.3–96.8	97.1		96.7	80.4–99.6	100	NA	100	NA

*ETF* early treatment failure, *LCF* late clinical failure, *LPF* late parasitological, *ACPR* adequate clinical and parasitological response

After PCR correction, ACPR rates of 97.8% (CI 95% 88.2–99.9) in Kelo and 100% in Doba (CI 95% 87.7–100) and in Kayoma (CI 95% 89.7–100), with an overall cure rate of 99.1% (CI 95% 94.9–100) were observed among AL treated patients (Table 4). For AS–AQ treated children, PCR corrected ACPR rate of 100% was observed in Doba (CI 95% 88.1–100), Kelo (CI 95% 88.8–100) and Kayoma (CI 95% 89.4–100), with overall cure rate of 100% (CI 95% 96.1–100). All, but one patient, were parasite free on day 3 (1/210, 0.5%).

**Table 4:** PCR-corrected treatment outcomes

Treatment Outcome	Artemether–lumefantrine						Artesunate-amodiaquine					
	Doba (n = 31)		Kelo (n = 48)		Kayom (n = 35)		Doba (n = 33)		Kelo (n = 33)		Kayom (n = 35)	
	n (%)	CI 95%	n (%)	95% CI	n (%)	95% CI	n (%)	CI 95%	n (%)	95% CI	n (%)	95% CI
*Per-protocol*												
ETF	0 (0)	0.0–12.3	0 (0)	0.0–7.9	0 (0)	0.0–10.3	0 (0)	0.0–11.9	0 (0)	0.0–11.2	0 (0)	0.0–10.6
LCF	0 (0)	0.0–12.3	0 (0)	0.0–7.9	0 (0)	0.0–10.3	0 (0)	0.0–11.9	0 (0)	0.0–11.2	0 (0)	0.0–10.6
LPF	0 (0)	0.0–12.3	1 (2.2)	0.1–11.8	0 (0)	0.0–10.3	0 (0)	0.0–11.9	0 (0)	0.0–11.2	0 (0)	0.0–10.6
ACPR	28 (100)	87.7–100	44 (97.8)	88.2–100	34 (100)	89.7–100	29 (100)	88.1–100	31 (100)	88.8–100	33 (100)	89.4–100
Total per-protocol	28		45		34		29		31		33	
Withdrawn/lost:	3 (9.7)		3 (6.3)		1 (2.9)		4 (12.1)		2 (6.1)		2 (5.7)	
Re-infection	–		3 (6.3)		–		–		–		–	
*Kaplan–Meier*												
Cure rate	100	NA	97.9	86.1–99.7	100	NA	100	NA	100	NA	100	NA

*ETF* early treatment failure, *LCF* late clinical failure, *LPF* late parasitological, *ACPR* adequate clinical and parasitological response

Both *msp1/mps2/polyα and msp1/msp2/glurp* genotyping methods gave similar results, as reinfection in five recurrent infections and one recurrent case was classified as recrudescence. The unique recrudescence case (AS556) was observed at day 7. The patient was a female of 4 years aged treated with AL. The doses given were supervised and were fully administered without side effects.

### Molecular markers

Out of the 215 day 0 samples analysed for the presence of polymorphism in *pfkelch13* gene, majority (96.7%) showed wild type allele and seven carried nonsynonymous mutations: the A578S was observed in 3 isolates (once in each site) known to not confer in vitro ART-R and the N489Y that is a non-validated *pfkelch13* mutation in 4 isolates (2 in Kelo and 2 in Koyom) (Table 5).

**Table 5:** Number and frequency of SNPs detected in genes associated with anti-malarial drug resistance in day0 samples by study site

Gene	Codon	AA	No. of sample				% of mutant (%)				p-value
			Doba	Kelo	Koyom	Total	Doba	Kelo	Koyom	Total	
*Kelch13*	WT	–	63	78	67	208	1.5	3.7	4.2	3.2	0.4
	N489	489T	0	2	2	4					
	A578	578S	1	1	1	3					
*Pfcrt*	72	C	64	81	70	215	0	0	0	0	–
		S	0	0	0	0					
	74	M	57	67	61	185	10.9	17.2	12.8	13.9	0.5
		I	7	14	9	30					
	75	N	57	67	61	185	10.9	17.2	12.8	13.9	0.5
		E	7	14	9	30					
	76	K	57	67	61	185	10.9	17.2	12.8	13.9	0.5
		T	7	14	9	30					
	93	T	64	81	70	215	0	0	0	0	–
		S	0	0	0	0					
	97	H	64	81	70	215	0	0	0	0	–
		Y	0	0	0	0					
	145	F	64	81	70	215	0	0	0	0	–
		I	0	0	0	0					
	218	I	64	75	68	207	0	7.4	2.8	3.7	0.2
		M	0	4	0	8					
		T	0	1	1						
		V	0	1	1						
	343	M	64	81	70	215	0	0	0	0	–
		L	0	0	0	0					
	350	C	64	81	70	215	0	0	0	0	–
		R	0	0	0	0					
	353	G	64	81	70	215	0	0	0	0	–
		V	0	0	0	0					
	356	I	60	72	63	195	6.2	11.1	10.0	9.3	0.6
		T	4	9	7	20					
*Pfmdr-1*	86	N	60	68	61	189	6.2	16.0	12.8	12.0	0.2
		Y	4	13	9	26					
	184	Y	21	29	25	75	67.1	64.1	64.2	65.11	0.9
		F	43	52	45	140					
	970	F	61	76	69	206	4.6	6.1	1.4	4.1	0.5
		I	1	1	1	3					
		L	0	2	0	2					
		S	0	1	0	1					
		V	2	1	0	3					
	1034	S	64	81	70	215	0	0	0	0	–
		C	0	0	0	0					
	1042	N	64	81	70	215	0	0	0	0	–
		D	0	0	0	0					
	1246	D	64	81	70	215	0	0	0	0	–
		Y	0	0	0	0					
*dhfr*	16	A	64	81	7	152	0	0	0	0	–
		V	0	0	0	0					
	50	C	64	81	7	152	0	0	0	0	–
		R	0	0	0	0					
	51	N	8	13	9	30	87.5	83.9	87.1	86.0	0.8
		I	56	68	61	185					
	59	C	8	6	7	21	87.5	92.5	90.0	90.2	0.6
		R	56	75	63	194					
	108	S	7	3	4	14	89.0	96.2	94.2	93.4	0.2
		N	57	78	66	201					
	164	I	64	81	7	152	0	0	0	0	–
		L	0	0	0	0					
*dhps*	431	I	53	71	60	184	17.1	12.3	14.2	14.4	0.7
		V	11	10	10	31					
	436	S	8	13	20	41	87.5	83.9	71.4	80.9	0.04
		A	56	68	50	174					
	437	G	26	23	36	85	59.3	71.6	48.5	60.4	0.01
		A	38	58	34	130					
	540	K	64	79	70	213	0	2.4	0	0.9	0.2
		E	0	2	0	2					
	581	A	64	81	7	152	0	0	0	0	–
		G	0	0	0	0					
	613	A	63	81	68	212	1.5	0	2.8	1.4	–
		S	1	0	2	3					

For *pfcrt,* eight alleles were detected, most of which were wild type (79% to 89%). The second most frequent alleles were alleles carrying the 74I/75E/76T mutations (4.7%) and the 74I/75E/76T/356T mutations (6.5%). The other mutations observed were at codons 145 (I> M or V). The most prevalent *pfmdr-1* allele detect was the single mutant 184F (51.2%, ranging from 48.6 to 60.9%) followed by the wild type allele (32.1%), double mutants 86Y/184F (8.8%) and the 184F/1034C (3.7%). The frequency of the other alleles was < 2%.

The triple mutant 51I/59R/108N was the most frequent *pfdhfr* allele (81.4% ranging from 79.7 to 84.3%), followed by the double mutant 59R/108N (7.0%), the wild type allele (4.7%) and the double mutant 51I/108N (3.3%). The frequency of the other alleles was < 2% and the quadruple mutant 51I/59R/108N/164L was absent. For *pfdhps*, the most frequent allele was the double mutant 436A/437A (54.7%, ranging from 47.1 to 62.5%), followed by the single mutant 437K (16.8%), the triple mutant 431V/436A/437K (11.7%), the double mutant 436A/437K (9.8%) and the triple mutant 431V/436A/437A (2.8%). The frequency of the other alleles was < 2%. The wild type allele and the 581G mutation were not detected. A significant difference in proportion were observed between sites (p = 0.04).

The most frequent *pfdhfr/pfdhps* alleles were the quintuple 51I/59R/108N/436A/437A mutant (44.7%), the quadruple 51I/59R/108N/437K (12.6%) mutant, the sextuple 51I/59R/108N/431V/436A/437K and 51I/59R/108N/431V/436A/437A mutants (11.2% and 2.8%, respectively); Lower proportions were observed for the quadruple 59R/108N/436A/437A (4.2%) mutant and the double 436A/437A mutant (3.3%). The proportions of the other alleles were < 2%. The quintuple mutant alleles N51I/C59R/S108N + G437A/540E responsible for SP treatment failures in adults and children (but still effective for use in intermittent preventive treatment in pregnancy) was not detected (Table 6).

**Table 6:**
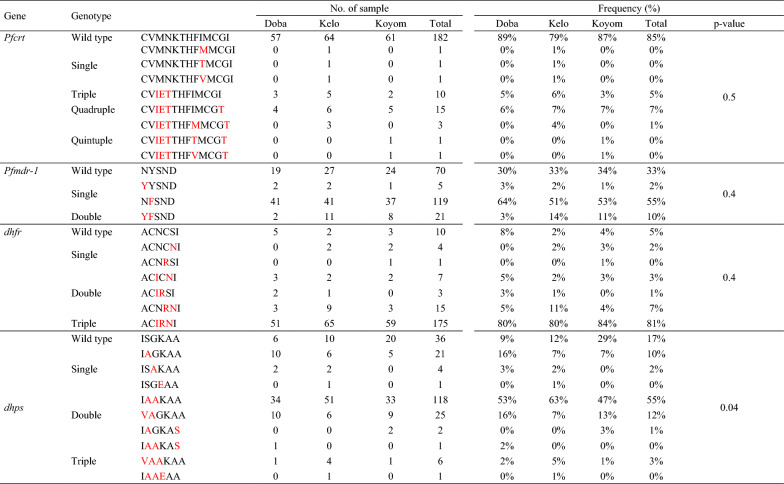
Number and frequency of different genotypes detected in genes associated with anti-malarial drug resistance from day0 samples by study site

Genetic deletions in the *hrp2* and *hrp3* genes in Chadian *P. falciparum* population were also sought as there are two important factors responsible for variable performance of malaria Rapid Diagnostic Tests. For this analysis, 215 day 0 samples were tested. Single deletion in the *hrp2* and *hrp3* gene were detected in 10/215 (4.7%, CI 95% 2.2–8.5) and 2/215 (0.9%, CI 95% 0.1–3.3), respectively. Dual *hrp2/hrp3* deletions, potentially threatening the efficacy of HRP2-based RDTs, were observed in 5/215 (2.3%, CI 95% 0.7–5.4) isolates in all sites: 2/64 (3.1%, CI 95% 0.4–1.1) in Doba, 2/81 (1.2%, CI 95% 0.03–6.8) in Kelo and 1/70 (1.4%, CI 95% 0.03–7.9) in Koyom.

## Discussion

The study revealed high cure rate (100%) of first-line treatment (AS–AQ) for uncomplicated malaria in children, confirming that ACT remains highly efficacious after its introduction in 2005. A similar high cure rate with AS–AQ was observed in 2015 [21]. The study also showed high efficacy (> 98%) of the second-line treatment (AL), which was evaluated for the first time since its recommendation in 2005. These findings are similar to the efficacy rates observed in neighboring Sudan [29], Central African Republic [30, 31], Cameroon [31], Nigeria [32], Niger [33, 34], and other West African countries [35–42]. A study in Burkina Faso reported a PCR-corrected cure rate of AL and DHA–PPQ of less than 80% at two sites [43]. One of the limitations of the study was that children weighing 5–9 kg received only half the intended doses. Other recent studies also reported AL cure rates below 90% in Angola [44] and the Democratic Republic of Congo [45] and may raise concern about the efficacy of the partner drug, lumefantrine. However, these studies used microsatellite-based PCR correction and a Bayesian algorithm for analysis, a methodological deviation from the standard WHO genotyping protocol, that has been associated with higher treatment failure in areas of high transmission [25]. Until a robust and validated tool to distinguish recrudescence from new infection is developed, therapeutic efficacy studies based on the standard WHO protocol [25] should be used to capture temporal comparability of efficacy data within and across countries.

Molecular analysis for polymorphism in the *pfkelch13* gene did not reveal any mutations known to be associated with delayed parasite clearance in Africa like the R561H, A675V or C469Y variants [6, 7]. Together with the high parasite clearance rate at day 3 (99.5%), this supports the absence of ART-R in the study areas, which is consistent with previous study [21]. However, the recent reports of validated *pfkelch13* mutations with delayed parasite clearance after ACT treatment in Rwanda [7], and Uganda [6] underscore the importance of continuing to monitor anti-malarial drug efficacy and resistance of artemisinins and partner drugs.

Molecular analysis of the current study revealed a low frequency of the *pfdhfr* wild-type allele (4.7%) with a high frequency of the triple mutant (51I/59R/108N) (81.4%) and the absence of the quintuple mutant alleles (N51I/C59R/S108N + G437A/540E). In contrast, a *pfdhfr* wild type of 53.3% and a very low frequency (3.3%) of the triple mutant (51I/59R/108N) were detected in the Pala West region in 2015 [21]. This difference could be due to either spatial or temporal differences, as the two studies were conducted in different areas and at different times. Overall, the absence of the quintuple and quintuple + 581G sextuple mutations, which have been associated with reduced efficacy of SP [12, 13] and decreased effectiveness of IPT-SP, respectively, in pregnant women [17, 21, 46], argues for the continued use of IPTp-SP. Studies from other West African countries reported the absence or very low frequency of the quintuple mutation [47–49]. Notably, this study reports for the first time the pfdhps I431V mutation, which was observed in proportions ranging from 12.3% (Kelo) to 17.1% (Doba) in the three study sites. This confirms previous molecular studies showing high levels of this mutation in Nigeria, Cameroon and other parts of West Africa. It is not yet clear whether this mutation interferes with the binding of sulfadoxine to its active site, thereby reducing the susceptibility of *P. falciparum* to the drug. However, epidemiological data show that the molecular repertoire of pfdhps in West Africa is clearly different from that in East Africa, where the K540E mutation remains low, while the I431V mutation appears to be emerging. More information is needed to assess the impact of haplotypes containing the I431V mutation on the protective efficacy of IPTp.

Most of the parasites carried the *pfcrt* wild type allele (79% to 89%). The proportion of *pfcrt* variant associated with resistance to chloroquine (CVIET, 11.2%) remains similar to previous report in 2016 (8/191, 4.2%) [50] and relatively low compared to other regions such as Ethiopia [51], Democratic Republic of the Congo [52], Equatorial Guinea [53], and Liberia [38]. For *pfmdr-1* gene, the single 184F mutation, which is suspected to be involved in recrudescent infections after AL treatment, was highly frequent in Chad as usually reported in numerous African settings such as Ghana [54], Uganda [55], Madagascar [56], Tanzania [57], and Equatorial Guinea [58].

Although, the study did not follow the recommended master protocol developed by WHO to guide surveillance and biobanking of *pfhrp2/3* gene deletions in malaria endemic countries, this study provides, for the first time, the overall prevalence of *hrp2*, *hrp3* and dual *hrp2/3* gene deletions estimated in some sites in Chad and baseline data for future surveys aimed at determining the trend in the frequency of *hrp2/3* gene deletions. The proportion of parasites with dual *hrp2/3* deletions found here (2.3%) is below the WHO 5% prevalence criterion defined for switching away from HRP2-based RDTs for malaria case management [59]. This frequency remains well below the proportions detected in the Horn of Africa such as Eritrea, Ethiopia, Djibouti, Sudan and South Sudan [60–63] and similar to proportions usually observed in African countries.

A limitation of the study is that the target sample size per drug at each site to detect treatment failure of 5% at a 95% CI and power of 10% could not be achieved because the study was conducted late in the malaria transmission season. Consequently, this study was underpowered to rule out low efficacy of the drugs tested.”

## Conclusion

The results of this study confirm that first-line (AS–AQ) and second line (AL) treatments are highly efficacious in the treatment of uncomplicated falciparum infections in Chad, with a cure rate of over 98%. The absence of known *pfkelch13* mutations in the study sites and the high parasite clearance rate at day 3 confirm the absence of ART-R. The absence of *pfdhfr/pfdhps* quintuple and quintuple + 581G sextuple mutations supports the continued use of SP for IPTp during pregnancy. Routine monitoring of anti-malarial drug efficacy and resistance should continue in Chad to detect any change in the susceptibility of the parasite populations.

### Supplementary Information

**Additional file 1: Table S1.** Raw data of *msp-1, msp-2*, *glurp* and *poly α* polymorphisms (band size in bp) detected on day0 and day of recurrence (dayX) in isolates from recurrent infections.

## Data Availability

The dataset used in this study is available and can be shared upon reasonable request to NMCP through the corresponding author.

## Abbreviations

ACPR: Adequate clinical and parasitological response
ACTs: Artemisinin-based combination therapies
AL: Artemether–lumefantrine
ART-R: Artemisinin resistance
AS–AQ: Artesunate–amodiaquine
AS–MQ: Artesunate–mefloquine
AS–PY: Artesunate–pyronaridine
AS–SP: Artesunate–sulfadoxine/pyrimethamine
*pfdhps*: *Plasmodium falciparum dihydropteroate synthetase*
DHA–PPQ: Dihydroartemisinin–piperaquine
ETF: Early treatment failure
LCF: Late clinical failure
LPF: Late parasitological failure
*msp1*: *Merozoite surface proteins 1*
*msp2*: *Merozoite surface proteins 2*
*glurp*: *Glutamate rich-protein*
PCR: Polymerase chain reaction
*pfdhfr*: *Plasmodium falciparum dihydrofolate reductase-thymidylate synthase*
*pfmdr-1*: *Plasmodium falciparum multi drug resistance 1*
*pfkelch13*: *Plasmodium falciparum kelch13*
WHO: World Health Organization

## References

[1] WHO (2023). Guidelines for malaria.

[2] WHO (2021). World malaria Report 2021.

[3] Ashley EA, Dhorda M, Fairhurst RM, Amaratunga C, Lim P, Suon S (2014). Spread of artemisinin resistance in *Plasmodium falciparum* malaria. N Engl J Med.

[4] Dondorp AM, Nosten F, Yi P, Das D, Phyo AP, Tarning J (2009). Artemisinin resistance in *Plasmodium falciparum* malaria. N Engl J Med.

[5] Ariey F, Witkowski B, Amaratunga C, Beghain J, Langlois AC, Khim N (2014). A molecular marker of artemisinin-resistant *Plasmodium falciparum* malaria. Nature.

[6] Balikagala B, Fukuda N, Ikeda M, Katuro OT, Tachibana SI, Yamauchi M (2021). Evidence of artemisinin-resistant malaria in Africa. N Engl J Med.

[7] Uwimana A, Legrand E, Stokes BH, Ndikumana JM, Warsame M, Umulisa N (2020). Emergence and clonal expansion of in vitro artemisinin-resistant *Plasmodium falciparum* kelch13 R561H mutant parasites in Rwanda. Nat Med.

[8] WHO (2020). Report on antimalarial drug efficacy, resistance and response: 10 years of surveillance (2010–2019).

[9] Lozovsky ER, Chookajorn T, Brown KM, Imwong M, Shaw PJ, Kamchonwongpaisan S (2009). Stepwise acquisition of pyrimethamine resistance in the malaria parasite. Proc Natl Acad Sci USA.

[10] Plowe CV, Cortese JF, Djimde A, Nwanyanwu OC, Watkins WM, Winstanley PA (1997). Mutations in *Plasmodium falciparum* dihydrofolate reductase and dihydropteroate synthase and epidemiologic patterns of pyrimethamine-sulfadoxine use and resistance. J Infect Dis.

[11] Triglia T, Menting JG, Wilson C, Cowman AF (1997). Mutations in dihydropteroate synthase are responsible for sulfone and sulfonamide resistance in *Plasmodium falciparum*. Proc Natl Acad Sci USA.

[12] Happi CT, Gbotosho GO, Folarin OA, Akinboye DO, Yusuf BO, Ebong OO (2005). Polymorphisms in *Plasmodium falciparum* dhfr and dhps genes and age related in vivo sulfadoxine-pyrimethamine resistance in malaria-infected patients from Nigeria. Acta Trop.

[13] Kublin JG, Dzinjalamala FK, Kamwendo DD, Malkin EM, Cortese JF, Martino LM (2002). Molecular markers for failure of sulfadoxine-pyrimethamine and chlorproguanil-dapsone treatment of *Plasmodium falciparum* malaria. J Infect Dis.

[14] Braun V, Rempis E, Schnack A, Decker S, Rubaihayo J, Tumwesigye NM (2015). Lack of effect of intermittent preventive treatment for malaria in pregnancy and intense drug resistance in western Uganda. Malar J.

[15] Gutman J, Kalilani L, Taylor S, Zhou Z, Wiegand RE, Thwai KL (2015). The A581G mutation in the gene encoding *Plasmodium falciparum* dihydropteroate synthetase reduces the effectiveness of sulfadoxine-pyrimethamine preventive therapy in Malawian pregnant women. J Infect Dis.

[16] Harrington WE, Mutabingwa TK, Kabyemela E, Fried M, Duffy PE (2011). Intermittent treatment to prevent pregnancy malaria does not confer benefit in an area of widespread drug resistance. Clin Infect Dis.

[17] Harrington WE, Mutabingwa TK, Muehlenbachs A, Sorensen B, Bolla MC, Fried M (2009). Competitive facilitation of drug-resistant *Plasmodium falciparum* malaria parasites in pregnant women who receive preventive treatment. Proc Natl Acad Sci USA.

[18] Plowe CV (2022). Malaria chemoprevention and drug resistance: a review of the literature and policy implications. Malar J.

[19] Chad National Strategic Plan for Malaria Control 2019–2023. https://scholar.google.com/citations?user=g5-0ZQwAAAAJ)

[20] Ministère de la Sante Publique République du Tchad. Programme National de Lutte Contre le Paludisme. N’Djamena, Chad, 2014.

[21] Issa MS, Ako AB, Kerah HC, Djimadoum M, Coulibaly B, Mbaitoloum MD, et al. Therapeutic efficacy of artesunate-amodiaquine and polymorphism of *Plasmodium falciparum* k13-propeller gene in Pala (Tchad). Int J Open Access Clin Trials 2017;1(1):1–6. https://symbiosisonlinepublishing.com/clinical-trials/clinical-trials04.pdf.

[22] WHO (2009). Methods for surveillance of antimalarial drug efficacy.

[23] Zainabadi K, Nyunt MM, Plowe CV (2019). An improved nucleic acid extraction method from dried blood spots for amplification of *Plasmodium falciparum* kelch13 for detection of artemisinin resistance. Malar J.

[24] Anderson TJ, Su XZ, Bockarie M, Lagog M, Day KP (1999). Twelve microsatellite markers for characterization of *Plasmodium falciparum* from finger-prick blood samples. Parasitology.

[25] World Health Organization (2021). Informal consultation on methodology to distinguish reinfection from recrudescence in high malaria transmission areas, 7–18 May 2021.

[26] WHO. Methods and techniques for clinical trials on antimalarial drug efficacy: genotyping to identify parasite populations: informal consultation organized by the Medicines for Malaria Venture and cosponsored by the World Health Organization, 29–31 May 2007, Amsterdam, The Netherlands. Geneva: World Health Organization; 2007.

[27] Menard D, Dondorp A (2017). Antimalarial drug resistance: a threat to malaria elimination. Cold Spring Harb Perspect Med.

[28] WHO (2019). False-negative RDT results and implications of new reports of *P. falciparum* histidine-rich protein 2/3 gene deletions.

[29] Adeel AA, Elnour FA, Elmardi KA, Abd-Elmajid MB, Elhelo MM, Ali MS (2016). High efficacy of artemether–lumefantrine and declining efficacy of artesunate + sulfadoxine–pyrimethamine against *Plasmodium falciparum* in Sudan (2010–2015): evidence from in vivo and molecular marker studies. Malar J.

[30] Nambei WS, Biago U, Balizou O, Pounguinza S, Moyen M, Ndoua C (2021). [Monitoring the efficacy of artemether–lumefantrine in the treatment of uncomplicated *Plasmodium falciparum* malaria by kelch 13 gene mutations in Bangui, Central African Republic]. Med Trop Sante Int.

[31] Niba PTN, Nji AM, Ali IM, Akam LF, Dongmo CH, Chedjou JPK (2022). Effectiveness and safety of artesunate-amodiaquine versus artemether–lumefantrine for home-based treatment of uncomplicated *Plasmodium falciparum* malaria among children 6–120 months in Yaounde, Cameroon: a randomized trial. BMC Infect Dis.

[32] Ebenebe JC, Ntadom G, Ambe J, Wammanda R, Jiya N, Finomo F (2018). Efficacy of artemisinin-based combination treatments of uncomplicated falciparum malaria in under-five-year-old Nigerian children ten years following adoption as first-line antimalarials. Am J Trop Med Hyg.

[33] Grandesso F, Guindo O, Woi Messe L, Makarimi R, Traore A, Dama S (2018). Efficacy of artesunate-amodiaquine, dihydroartemisinin-piperaquine and artemether–lumefantrine for the treatment of uncomplicated *Plasmodium falciparum* malaria in Maradi, Niger. Malar J.

[34] Ibrahima I, Laminou IM, Adehossi E, Maman D, Boureima S, Harouna HK (2020). Safety and efficacy of artemether–lumefantrine and artesunate-amodiaquine in Niger. Bull Soc Pathol Exot.

[35] Abuaku B, Duah-Quashie NO, Quaye L, Matrevi SA, Quashie N, Gyasi A (2019). Therapeutic efficacy of artesunate-amodiaquine and artemether–lumefantrine combinations for uncomplicated malaria in 10 sentinel sites across Ghana: 2015–2017. Malar J.

[36] Beavogui AH, Camara A, Delamou A, Diallo MS, Doumbouya A, Kourouma K (2020). Efficacy and safety of artesunate-amodiaquine and artemether–lumefantrine and prevalence of molecular markers associated with resistance, Guinea: an open-label two-arm randomised controlled trial. Malar J.

[37] Diallo MA, Yade MS, Ndiaye YD, Diallo I, Diongue K, Sy SA (2020). Efficacy and safety of artemisinin-based combination therapy and the implications of Pfkelch13 and Pfcoronin molecular markers in treatment failure in Senegal. Sci Rep.

[38] Koko VS, Warsame M, Vonhm B, Jeuronlon MK, Menard D, Ma L (2022). Artesunate-amodiaquine and artemether–lumefantrine for the treatment of uncomplicated falciparum malaria in Liberia: in vivo efficacy and frequency of molecular markers. Malar J.

[39] Konate A, Barro-Kiki PCM, Angora KE, Bedia-Tanoh AV, Djohan V, Kassi KF (2018). Efficacy and tolerability of artesunate-amodiaquine versus artemether–lumefantrine in the treatment of uncomplicated *Plasmodium falciparum* malaria at two sentinel sites across Cote d'Ivoire. Ann Parasitol.

[40] Lingani M, Bonkian LN, Yerbanga I, Kazienga A, Valea I, Sorgho H (2020). In vivo/ex vivo efficacy of artemether–lumefantrine and artesunate-amodiaquine as first-line treatment for uncomplicated falciparum malaria in children: an open label randomized controlled trial in Burkina Faso. Malar J.

[41] Riloha Rivas M, Warsame M, Mba Andeme R, Nsue Esidang S, Ncogo PR, Phiri WP (2021). Therapeutic efficacy of artesunate-amodiaquine and artemether–lumefantrine and polymorphism in *Plasmodium falciparum* kelch13-propeller gene in Equatorial Guinea. Malar J.

[42] Smith SJ, Kamara ARY, Sahr F, Samai M, Swaray AS, Menard D (2018). Efficacy of artemisinin-based combination therapies and prevalence of molecular markers associated with artemisinin, piperaquine and sulfadoxine-pyrimethamine resistance in Sierra Leone. Acta Trop.

[43] Gansane A, Moriarty LF, Menard D, Yerbanga I, Ouedraogo E, Sondo P (2021). Anti-malarial efficacy and resistance monitoring of artemether–lumefantrine and dihydroartemisinin–piperaquine shows inadequate efficacy in children in Burkina Faso, 2017–2018. Malar J.

[44] Dimbu PR, Horth R, Candido ALM, Ferreira CM, Caquece F, Garcia LEA (2021). Continued low efficacy of artemether–lumefantrine in Angola in 2019. Antimicrob Agents Chemother.

[45] Moriarty LF, Nkoli PM, Likwela JL, Mulopo PM, Sompwe EM, Rika JM (2021). Therapeutic efficacy of artemisinin-based combination therapies in Democratic Republic of the Congo and Investigation of molecular markers of antimalarial resistance. Am J Trop Med Hyg.

[46] Chico RM, Cano J, Ariti C, Collier TJ, Chandramohan D, Roper C (2015). Influence of malaria transmission intensity and the 581G mutation on the efficacy of intermittent preventive treatment in pregnancy: systematic review and meta-analysis. Trop Med Int Health.

[47] Afutu LL, Boampong JN, Quashie NB (2021). High prevalence of molecular markers of *Plasmodium falciparum* resistance to sulphadoxine-pyrimethamine in parts of Ghana: a threat to ITPTp-SP?. J Trop Pediatr.

[48] Dicko A, Sagara I, Djimde AA, Toure SO, Traore M, Dama S (2010). Molecular markers of resistance to sulphadoxine-pyrimethamine one year after implementation of intermittent preventive treatment of malaria in infants in Mali. Malar J.

[49] Naidoo I, Roper C (2013). Mapping 'partially resistant', 'fully resistant', and 'super resistant' malaria. Trends Parasitol.

[50] Das S, Kerah-Hinzoumbe C, Kebfene M, Srisutham S, Nagorngar TY, Saralamba N (2022). Molecular surveillance for operationally relevant genetic polymorphisms in *Plasmodium falciparum* in Southern Chad, 2016–2017. Malar J.

[51] Hassen J, Alemayehu GS, Dinka H, Golassa L (2022). High prevalence of Pfcrt 76T and Pfmdr1 N86 genotypes in malaria infected patients attending health facilities in East Shewa zone, Oromia Regional State, Ethiopia. Malar J.

[52] Yobi DM, Kayiba NK, Mvumbi DM, Boreux R, Kabututu PZ, Akilimali PZ (2022). Biennial surveillance of *Plasmodium falciparum* anti-malarial drug resistance markers in Democratic Republic of Congo, 2017 and 2019. BMC Infect Dis.

[53] Berzosa P, Molina de la Fuente I, Ta-Tang TH, Gonzalez V, Garcia L, Rodriguez-Galet A (2021). Temporal evolution of the resistance genotypes of *Plasmodium falciparum* in isolates from Equatorial Guinea during 20 years (1999 to 2019). Malar J.

[54] Mensah BA, Aydemir O, Myers-Hansen JL, Opoku M, Hathaway NJ, Marsh PW (2020). Antimalarial drug resistance profiling of *Plasmodium falciparum* infections in Ghana using molecular inversion probes and next-generation sequencing. Antimicrob Agents Chemother.

[55] Tumwebaze PK, Katairo T, Okitwi M, Byaruhanga O, Orena S, Asua V (2021). Drug susceptibility of *Plasmodium falciparum* in eastern Uganda: a longitudinal phenotypic and genotypic study. Lancet Microbe.

[56] Dentinger CM, Rakotomanga TA, Rakotondrandriana A, Rakotoarisoa A, Rason MA, Moriarty LF (2021). Efficacy of artesunate-amodiaquine and artemether–lumefantrine for uncomplicated *Plasmodium falciparum* malaria in Madagascar, 2018. Malar J.

[57] Bwire GM, Ngasala B, Mikomangwa WP, Kilonzi M, Kamuhabwa AAR (2020). Detection of mutations associated with artemisinin resistance at k13-propeller gene and a near complete return of chloroquine susceptible falciparum malaria in Southeast of Tanzania. Sci Rep.

[58] Liu Y, Liang X, Li J, Chen J, Huang H, Zheng Y (2022). Molecular surveillance of artemisinin-based combination therapies resistance in *Plasmodium falciparum* parasites from Bioko Island, Equatorial Guinea. Microbiol Spectr.

[59] Menegon M, L'Episcopia M, Nurahmed AM, Talha AA, Nour BYM, Severini C (2017). Identification of *Plasmodium falciparum* isolates lacking histidine-rich protein 2 and 3 in Eritrea. Infect Genet Evol.

[60] Alemayehu GS, Messele A, Blackburn K, Lopez K, Lo E, Janies D (2021). Genetic variation of *Plasmodium falciparum* histidine-rich protein 2 and 3 in Assosa zone, Ethiopia: its impact on the performance of malaria rapid diagnostic tests. Malar J.

[61] Golassa L, Messele A, Amambua-Ngwa A, Swedberg G (2020). High prevalence and extended deletions in *Plasmodium falciparum* hrp2/3 genomic loci in Ethiopia. PLoS ONE.

[62] Iriart X, Menard S, Chauvin P, Mohamed HS, Charpentier E, Mohamed MA (2020). Misdiagnosis of imported falciparum malaria from African areas due to an increased prevalence of pfhrp2/pfhrp3 gene deletion: the Djibouti case. Emerg Microbes Infect.

[63] Prosser C, Gresty K, Ellis J, Meyer W, Anderson K, Lee R (2021). *Plasmodium falciparum* histidine-rich protein 2 and 3 gene deletions in strains from Nigeria, Sudan, and South Sudan. Emerg Infect Dis.

