# Supplementary material for: Therapeutic efficacy of artesunate–amodiaquine and artemether–lumefantrine for the treatment of uncomplicated falciparum malaria in Chad: clinical and genetic surveillance
Source: Malar J. 2023 Aug 23;22:240. doi: 10.1186/s12936-023-04644-w (PMC10464190; doi:10.1186/s12936-023-04644-w)
Supplement: Supplementary file 1 — Additional file 1: Table S1. Raw data of msp-1, msp-2, glurp and poly α polymorphisms (band size in bp) detected on day0 and day of recurrence (dayX) in isolates from recurrent infections. [file 12936_2023_4644_MOESM1_ESM.docx]

Table S1. Raw data of *msp-1, msp-2*, *glurp* and *poly α* polymorphisms (band size in bp) detected on day0 and day of recurrence (dayX) in isolates from recurrent infections.

| ID | Day0 | Dayx | MSP1 | | | | | | MSP2 | | | | GLURP | | poly α | |
| --- | --- | --- | --- | --- | --- | --- | --- | --- | --- | --- | --- | --- | --- | --- | --- | --- |
|  |  |  | K1 D0 | K1 DX | RO33 D0 | RO33 DX | Mad20 D0 | Mad20 DX | 3D7 D0 | 3D7 DX | FC27 D0 | FC27 DX | glurp D0 | glurp DX | poly α D0 | poly α DX |
| NF611 | Day 0 | Day 28 | - | - | 135 | 135 | 150 | - | 250/300 | - | 510 | 400 | 650/800/900 | 700 | 180 | 180 |
| GK336 | Day 0 | Day 28 | 210 | 180 | - | - | 180 | 180 | 310 | 290 | 380/400 | 480 | 700 | 800 | 180 | 180 |
| BC341 | Day 0 | Day 21 | 170/200 | 170 | 135 | - | 180/220/250 | 180 | 280/350/410 | 380 | 420 | 480 | 700 | 700 | 180 | 180 |
| BC348 | Day 0 | Day 28 | - | - | - | - | - | 170 | 250 | 380 | - | - | 700 | 800 | 160 | 180 |
| AS556 | Day 0 | Day 7 | 210 | 210 | - | - | 200 | 200 | 380 | 380 | 380 | 380 | 800 | 800 | 160 | 160 |
| AI338 | Day 0 | Day 28 | - | - | - | 135 | - | - | - | - | 450 | 380 | 650 | 700 | 200 | - |
| MA280 | Day 0 | Day 28 | - | - | - | - | 210 | 210 | - | - | - | 500 | - | - | 160 | - |

| ID | Day0 | Dayx | MSP1 | MSP2 | Glurp | poly α | Conclusion | |
| --- | --- | --- | --- | --- | --- | --- | --- | --- |
|  |  |  |  |  |  |  | msp1/msp2/glurp | msp1/msp2/poly α |
| NF611 | Day 0 | Day 28 | Recrudescence | Reinfection | Reinfection | Recrudescence | Reinfection | Reinfection |
| GK336 | Day 0 | Day 28 | Recrudescence | Reinfection | Reinfection | Recrudescence | Reinfection | Reinfection |
| BC341 | Day 0 | Day 21 | Recrudescence | Reinfection | Recrudescence | Recrudescence | Reinfection | Reinfection |
| BC348 | Day 0 | Day 28 | NI | Reinfection | Reinfection | Reinfection | Reinfection | Reinfection |
| AS556 | Day 0 | Day 7 | Recrudescence | Recrudescence | Recrudescence | Recrudescence | Recrudescence | Recrudescence |
| AI338 | Day 0 | Day 28 | NI | Reinfection | Reinfection | NI | Reinfection | Reinfection |
| MA280 | Day 0 | Day 28 | Recrudescence | NI | NI | NI | Recrudescence | Recrudescence |
